# Unraveling Kinase Activation Dynamics Using Kinase-Substrate Relationships from Temporal Large-Scale Phosphoproteomics Studies

**DOI:** 10.1371/journal.pone.0157763

**Published:** 2016-06-23

**Authors:** Westa Domanova, James Krycer, Rima Chaudhuri, Pengyi Yang, Fatemeh Vafaee, Daniel Fazakerley, Sean Humphrey, David James, Zdenka Kuncic

**Affiliations:** 1 Charles Perkins Centre, The University of Sydney, Sydney, NSW 2006, Australia; 2 School of Physics, The University of Sydney, Sydney, NSW 2006, Australia; 3 School of Life and Environmental Sciences, The University of Sydney, Sydney, NSW 2006, Australia; 4 National Institute of Environmental Health Sciences, National Institutes of Health, Research Triangle Park, Durham, NC 27709, United States of America; 5 School of Mathematics and Statistics, The University of Sydney, Sydney, NSW 2006, Australia; 6 Department of Proteomics and Signal Transduction, Max Planck Institute for Biochemistry, Martinsried, 82152, Germany; 7 Sydney Medical School, The University of Sydney, Sydney, NSW 2006, Australia; Huazhong University of Science and Technology, CHINA

## Abstract

In response to stimuli, biological processes are tightly controlled by dynamic cellular signaling mechanisms. Reversible protein phosphorylation occurs on rapid time-scales (milliseconds to seconds), making it an ideal carrier of these signals. Advances in mass spectrometry-based proteomics have led to the identification of many tens of thousands of phosphorylation sites, yet for the majority of these the kinase is unknown and the underlying network topology of signaling networks therefore remains obscured. Identifying kinase substrate relationships (KSRs) is therefore an important goal in cell signaling research. Existing consensus sequence motif based prediction algorithms do not consider the biological context of KSRs, and are therefore insensitive to many other mechanisms guiding kinase-substrate recognition in cellular contexts. Here, we use temporal information to identify biologically relevant KSRs from Large-scale In Vivo Experiments (KSR-LIVE) in a data-dependent and automated fashion. First, we used available phosphorylation databases to construct a repository of existing experimentally-predicted KSRs. For each kinase in this database, we used time-resolved phosphoproteomics data to examine how its substrates changed in phosphorylation over time. Although substrates for a particular kinase clustered together, they often exhibited a different temporal pattern to the phosphorylation of the kinase. Therefore, although phosphorylation regulates kinase activity, our findings imply that substrate phosphorylation likely serve as a better proxy for kinase activity than kinase phosphorylation. KSR-LIVE can thereby infer which kinases are regulated within a biological context. Moreover, KSR-LIVE can also be used to automatically generate positive training sets for the subsequent prediction of novel KSRs using machine learning approaches. We demonstrate that this approach can distinguish between Akt and Rps6kb1, two kinases that share the same linear consensus motif, and provide evidence suggesting IRS-1 S265 as a novel Akt site. KSR-LIVE is an open-access algorithm that allows users to dissect phosphorylation signaling within a specific biological context, with the potential to be included in the standard analysis workflow for studying temporal high-throughput signal transduction data.

## Introduction

Cells use intricate signaling networks to monitor and respond to environmental cues and to appropriately regulate specialized biological functions such as differentiation, metabolism and proliferation. A significant portion of signal transduction is mediated via the posttranslational modification (PTM) of proteins. One of the most prevalent and acute PTMs is phosphorylation, particularly on Ser/Thr residues. Phosphorylation is mediated by protein kinases, each of which targets a specific subset of protein substrates. The specificity of these interactions is governed by a range of factors such as the structure of the kinase catalytic site, subcellular localization and the formation of regulatory scaffolds and adaptor proteins [1]. This specificity enables the cell to respond precisely to external stimuli.

The study of cell signaling networks has been revolutionized by high throughput proteomics methods and analytical workflows, enabling collection, analysis and quantification of protein phosphorylation on a global scale (hereafter called “phosphoproteomics”) [2]. Current large-scale phosphoproteomics experiments employing extensive fractionation can identify more than 30,000 phosphorylation sites [3], revealing that as many as two thirds of the proteins in the cell are phosphorylated [3,4]. In addition to being able to measure the phosphoproteome to great depth, recent developments now enable quantification of the phosphoproteome across hundreds of samples in a high-throughput and reproducible manner [5,6]. The availability of increasingly large volumes of phosphoproteomics data poses new challenges. Most notably, there is a growing need to identify the links between kinases and the thousands of phosphorylation sites identified in these studies. This will greatly help to map the structure of signaling networks, understanding which, when, and how kinases respond to different external cues.

A key development in identifying the relationships between kinases and their substrates was the recognition that short stretches of amino acid sequence (consensus sequence motifs) could be used to predict kinase-substrates [7]. This has been used as the foundation for numerous computational methods for predicting KSRs, including ScanSite [8], GPS [9], NetPhosK [10] and KinasePhos [11]. However this approach is limited by the fact that closely related kinases belonging to the same family often share highly similar phosphorylation recognition motifs. For example, several kinases of the AGC family (e.g. Akt, and S6K) recognize the same consensus motif RxRxxS/T [12]. Dissecting precisely which kinase is responsible for phosphorylating a substrate can therefore be particularly challenging especially if these related kinases also form part of the same signaling network [13]. Therefore, methods which utilize information in addition to linear sequence are required to improve prediction accuracy. One method that has been extensively used is integrating information about protein-protein interactions (PPI) and the consensus motif (e.g. NetworKIN [14], iGPS [15]). However, PPI databases (e.g. STRING [16]) typically have low information content about kinases and their substrates, since the transient interactions between these molecules are not captured by affinity-purification experiments. Moreover these data are derived from experiments performed under a wide range of different conditions including different cell lines and stimuli. Curating signaling networks in a manual or semi-automated manner using literature-derived knowledge can circumvent these caveats, however this is a time consuming process and is prone to false negatives, owing to a lack of high-quality supporting experimental data. In a study of dynamic phosphorylation in adipocytes, we found that the temporal change in phosphorylation of kinase substrates in response to a perturbation provides a high resolution method of segregating kinase activation [17]. We therefore propose that temporal information may serve to help identify kinases active under a specific biological context from large-scale phosphoproteomics data.

Here, we developed an approach to enable automatic identification of biologically relevant KSRs–substrates that are phosphorylated by a kinase within the biological context of the experiment–using Large scale In Vivo temporal Experiments (KSR-LIVE). KSR-LIVE sources previously reported experimentally-derived KSR data from a comprehensive knowledgebase, and uses a clustering procedure on temporal data to filter for biologically relevant KSRs. KSR-LIVE can be easily integrated into standard bioinformatics workflows, and is available as an R package on CRAN (*https://cran.r-project.org/package=ksrlive*).

## Results

We formulated an approach to identify biologically relevant KSRs in large-scale datasets in an automated fashion. First, the phosphoproteomics dataset is compared to a database of experimentally validated KSRs to extract potential substrates for each kinase. Next, the temporal profiles of these potential substrates are clustered, to identify biologically relevant substrates for each kinase within the dataset’s experimental context. This substrate list can then be utilized as markers for kinase activity and as a training set for predicting novel KSRs.

### Construction of a comprehensive knowledgebase of site-specific kinase substrate relationships

To generate a large source of experimentally derived KSRs, we combined the knowledge of four resources: PhosphoSitePlus [18], PhosphoELM [19], PhosphoPOINT [20] and Human Protein Reference Database (HPRD) [21]. Overall this integrated database contained 396 kinases, 76% coverage of the human kinome [22]. There were approximately 8,000 phosphorylation sites on > 2,000 proteins where the kinase was provided in the database, this resulted in approximately 11,500 KSRs. Of these KSRs, ~38% were uniquely found in PhosphoSitePlus, yet only 12% were found in all of the databases, justifying the integration of several data sources.

### Tight clustering enables identification of biologically relevant KSRs

As a case study for developing this approach we used a time-resolved phosphoproteome previously reported by our group [17]. This was derived from 3T3-L1 adipocytes stimulated with insulin for specific times, and is herein termed ‘Insulin Dataset’. For each kinase within the KSR knowledgebase, we searched for substrates within the Insulin Dataset. Out of the 5,873 regulated phosphosites in the Insulin Dataset, 456 were found in the knowledgebase, from which we identified potential substrates for 150 kinases. Substrates could have multiple kinases reported, with only some of these KSRs occurring within the experimental context.

Given that the phosphorylation state of a substrate reflects the activity of its corresponding kinase, we can examine substrate phosphorylation profiles to gauge how the activity of their kinase changes over time. Indeed, we previously observed that substrates of the same kinase are more likely to co-cluster [17]. Thus, we developed a method for identifying such clusters in an automated fashion.

Furthermore, not all substrates in the KSR database may be phosphorylated in the data, thus we can use these clusters to determine KSRs biologically relevant in the context of the dataset analyzed. To this end, we extracted substrates by subjecting the temporal profiles of substrates to tight clustering [23]. This clustering approach offers the advantage of identifying stable tight clusters, and is therefore robust to noisy measurements.

For each kinase, tight clustering was performed in two steps, on different subsets of the potential substrates. In the first step, we considered only ‘exclusive substrates’, substrates only reported to be phosphorylated by one kinase. The resulting tight clusters formed the ‘core substrates’ for the kinase. In the second step, tight clustering was performed on the time profiles of all the potential substrates in the data. All tight clusters containing the core substrates were subsequently taken as markers of kinase activity and form the characteristic temporal activation (CTA) profile for the kinase. For some kinases multiple exclusive clusters could be identified and in those cases all exclusive clusters were included in the analysis. The substrates that form the CTA are listed in S1 Table.

This two-step clustering procedure identifies the activation pattern for each kinase in an unbiased, automated fashion.

**Fig 1:**
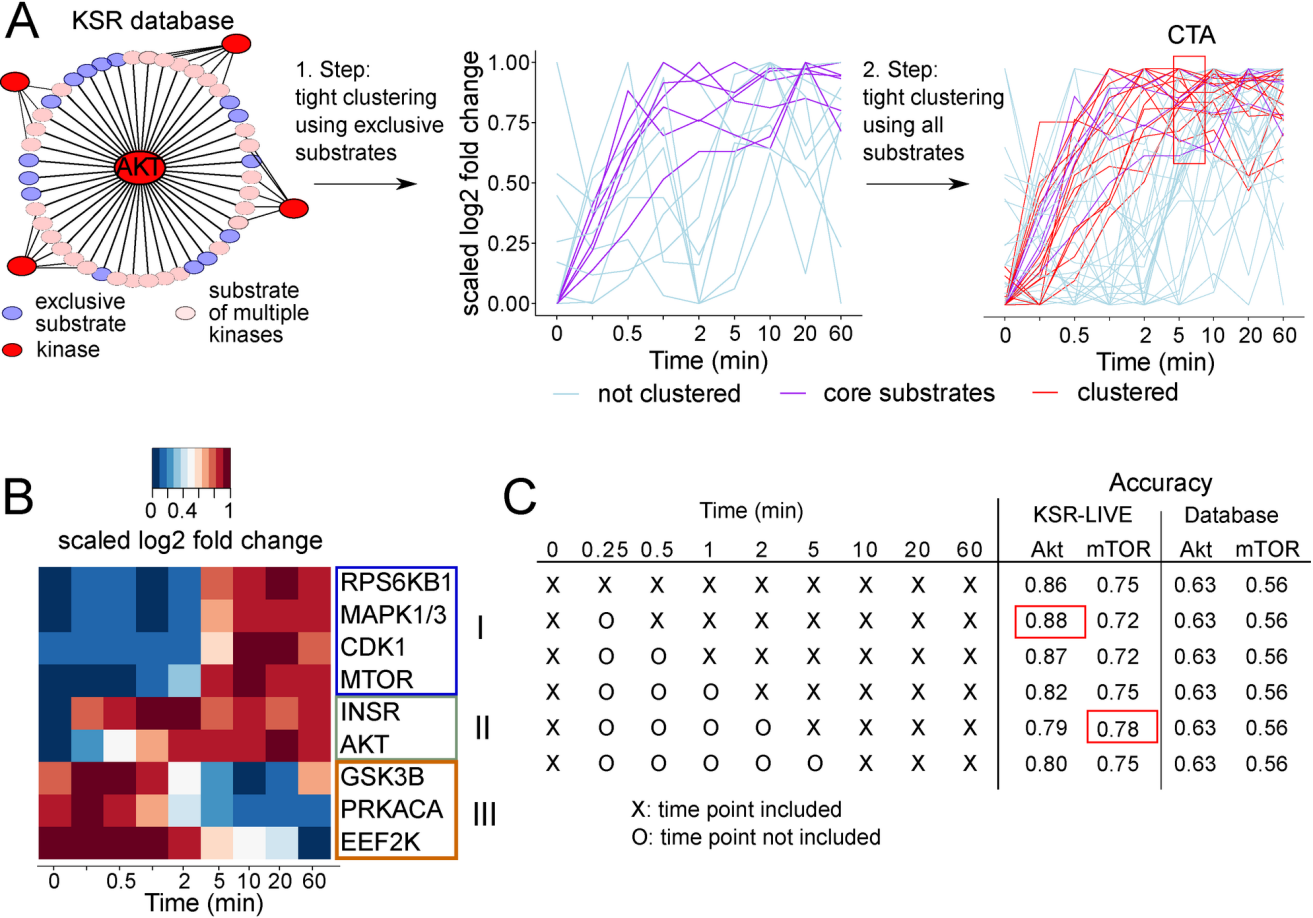
Overview of KSR-LIVE. A) Flowchart of clustering procedure. Substrates for a kinase (for example Akt) are extracted from the KSR knowledgebase and can either be exclusive (blue) or not (pink). In the first step tight clustering is performed on exclusive substrates and core substrates (purple) identified. In the second step tight clustering is performed using all substrates and the characteristic temporal activity of a kinase is identified. B) Heatmap of scaled log fold change of the characteristic temporal activity of 9 kinases over time. High log fold change is represented in red, low log fold change is shown in blue C) Table showing the time points included in the accuracy analysis and the accuracy of using a database or KSR-LIVE for Akt and mTOR.

As an example, we sourced substrates from our KSR knowledgebase for the kinase Akt, an intensely-studied kinase that is activated in response to insulin [24,25]. From the 222 Akt substrates found in the KSR knowledgebase, 44 were identified in the Insulin Dataset. The first clustering step identified 6 core substrates from 15 substrates that were exclusive to Akt (Fig 1A). Tight clustering with all potential substrates identified a single CTA consisting of 16 substrates (Fig 1A). The other substrates did not cluster in 90% of the resampling-based tight clustering runs (a cutoff recommended by the developers of the tight clustering algorithm [23]). The Akt CTA was rapid, saturating within 30 s, as reported previously [17]. The excluded potential substrates may involve additional regulation of their phosphorylation such as localization, may be phosphorylated by other kinases in this context, or may not have robust quantitative temporal profiles in the mass spectrometry data analyzed.

We next expanded our analysis to explore substrates for other kinases in the Insulin Dataset. To be included in the KSR-LIVE analysis, kinases had to have more than 2 exclusive substrates; 23 kinases fulfilled these criteria, and using KSR-LIVE, we extracted CTAs for 9 of these 23 kinases (Akt, Insr, Cdk1/2, mTOR, Mapk1/3, Rps6kb1, Gsk3b, Prkaca, Eef2k), all of which are implicated in insulin signaling [24,26–28]. No CTA profile could be found for the remaining 14 kinases, because a tight cluster could not be identified from the exclusive substrates. This can be attributed to kinases that do not respond robustly to insulin, or those which currently have insufficient numbers of known exclusive substrates in the KSR knowledgebase.

Among the kinases whose CTA profiles could be determined, their CTAs could be divided into three distinct groups based on their temporal responses (Fig 1B). Akt and Insr were early responders, being activated as early as 15 s after insulin addition. A slower group, consisting of mTOR, Mapk1/3, Rps6kb1 and Cdk1/2, were activated between 5–10 min after insulin addition. In contrast, substrates of Gsk3b, Prkaca and Eef2k displayed reduced phosphorylation upon insulin stimulation, indicating deactivation of these kinases. Gsk3b formed a special case as it was reactivated again after 20 min. It is important to note that although the time profiles of the individual substrates are not identical, the cluster time profiles can serve as a general reflection of kinase activity–for instance, a kinase activated early in a time-course is more likely to have its substrate cluster changing early as well. Thus, the temporal resolution of the Insulin Dataset enabled KSR-LIVE to identify distinct CTA profiles of several kinases in an unbiased fashion, without any prior knowledge of the kinases involved in insulin signaling.

### KSR-LIVE approximates manual curation of biochemically-validated KSRs

We compared the identified biologically relevant substrates to a standard reference. We chose substrates for Akt and mTOR [17] that had been manually curated previously as reference, mTOR substrates were used as the negative set for calculation of Akt accuracy and vice versa. The manually curated substrates for Akt and mTOR were selected giving preference towards KSRs with high-quality supporting data from reductionist-based biochemical studies, or for which there were multiple lines of evidence from different sources. The list of substrates created by KSR-LIVE and the manually curated lists of substrates shared good overlap, they had 14 substrates in common for Akt and 8 for mTOR (S1A Fig). Some substrates were exclusive to the manually curated lists because they were not included in the current databases, and therefore could not be extracted automatically. There were also substrates specific to the automated sets likely excluded from manual lists due to the limitation associated with the curator’s knowledge and/or to stringency of curation criteria. Nevertheless, the CTAs generated by manual and automatically generated training sets were remarkably similar, with a correlation coefficient of 0.97 between the means of the sets.

Further, we investigated the sensitivity of KSR-LIVE to the number of time points sampled in the phosphoproteomics data by removing time points one at a time, and comparing the identified CTA substrates to the manually curated substrates (Fig 1C). The KSR knowledgebase baseline, the Rand accuracy of the KSR knowledgebase without any temporal information was 56% for mTOR and 63% for Akt, indicating that the KSR knowledgebase suffers from a high number of false positives. Using KSR-LIVE to identify substrates increased the accuracy for both Akt and mTOR to over 70% for all numbers of time points. Therefore, additional temporal information significantly improves the accurate identification of high quality KSRs.

### Kinase phosphorylation temporal-profiles are often, but not always, similar to their CTAs

**Fig 2:**
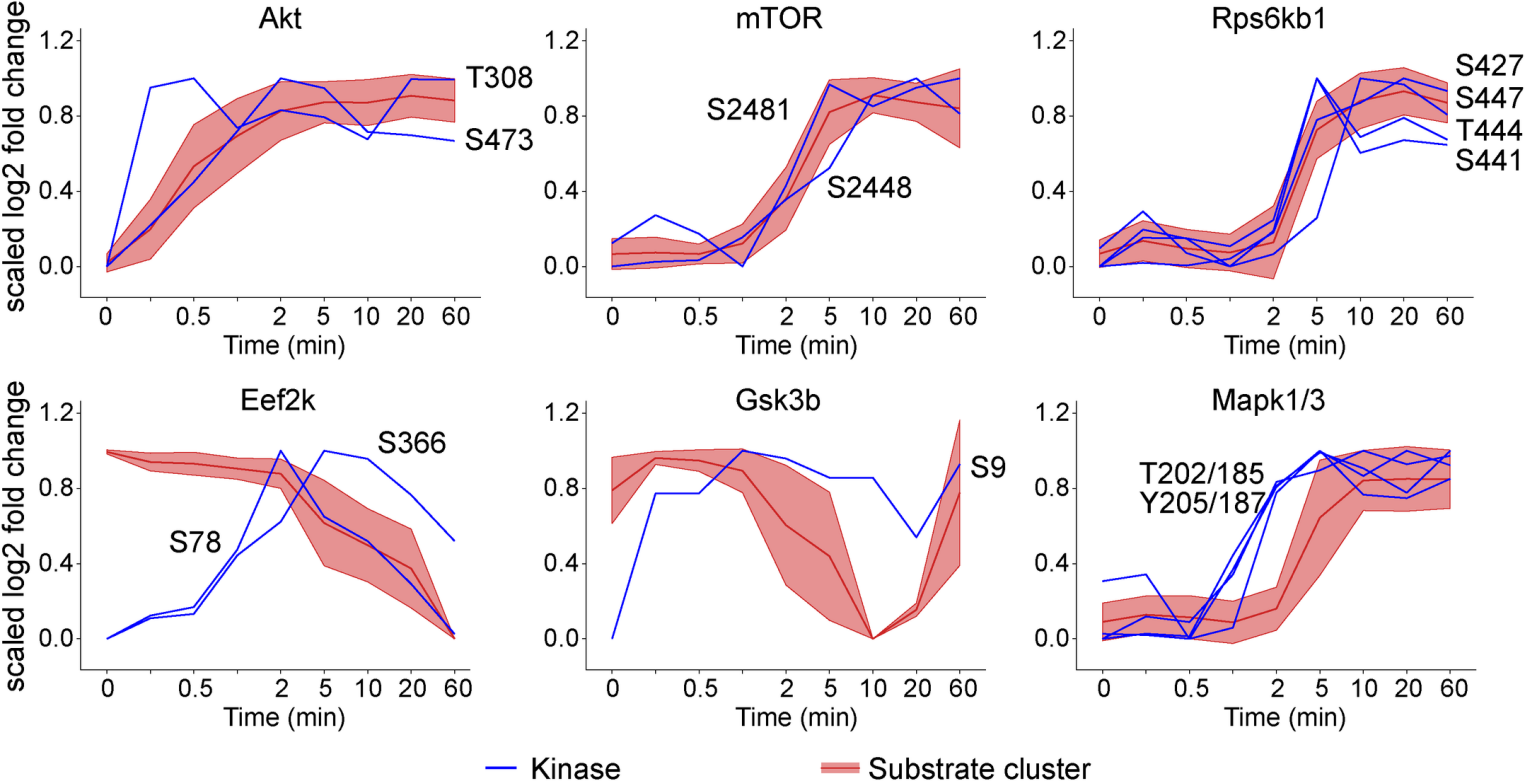
Scaled log fold change over time of kinase (shown in blue) and the corresponding CTA (shown in red, mean ± SD) for multiple kinases.

Kinases are often regulated by phosphorylation so kinase phosphorylation is often used as a proxy for activity. Thus, we compared the kinase CTAs to the phosphorylation profiles of the kinases themselves (Fig 2). We expected the CTA would either co-segregate with or precede kinase phosphorylation, the latter occurring if additional regulatory events were required to mediate kinase activation. Phosphorylation of mTOR and Rps6kb1 at S2481/S2448 and S427/S441/S447/T444, respectively followed a similar time profile as their substrates (Fig 2). In contrast, phosphorylation of Akt within its activation loop at T308 [29] occurred prior to phosphorylation on its S473 site and its substrates, consistent with the stepwise activation of Akt [13]. Mapk1/3 displayed a different pattern, with phosphorylation of the kinase at its key regulatory sites T202/185, Y205/187 preceding any detectable substrate phosphorylation by 1 min. For Eef2k, S78 phosphorylation decreases with the CTA.

In contrast, it is known that Eef2k and Gsk3b are deactivated upon phosphorylation at S78/366 and S9, respectively [30–32]. Within the Insulin Dataset, it is evident that the effect of phosphorylating these sites is not immediate, with the kinase CTA decreasing only when the inhibitory phosphorylation of Eef2k and Gsk3b reached their maximum. For Eef2k, S78 phosphorylation decreases with the CTA and for Gsk3B, the CTA profile increases after 10–20 min whilst kinase phosphorylation on S9 is maintained.

In conclusion, we observed instances where the temporal relationship of phosphorylation of the kinase does not align precisely with the phosphorylation of its substrates, suggesting that kinase CTA profiles are a more suitable marker of kinase activity than phosphorylation of kinases themselves.

### KSR-LIVE can be applied to other temporal phosphoproteomics data

We next applied KSR-LIVE to another recently published temporal phosphoproteomics dataset–a time-course of NGF stimulation in a human neuroblastoma cell line (SH-SY5Y) [33], hereafter referred to as ‘NGF Dataset’. The analysis of these data by the authors included fuzzy clustering, from which they were able to identify a cluster of early sustained responders (active after 10 min and throughout the experiment). They also used GO term enrichment to identify the involvement of Mitogen activated protein kinases (MAPKs) in NGF signaling.

**Fig 3:**
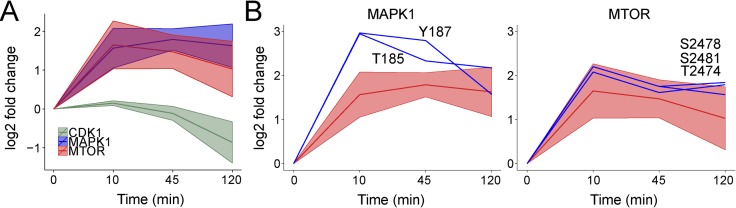
Analysis of Emdal *et al*. data using KSR-LIVE. A) Log fold change of MAPK1, MTOR and CDK1 CTAs (shown in red, mean ± SD). B) Log fold change of kinase phosphorylation (blue) and the corresponding CTA (shown in red, mean ± SD) for multiple kinases.

KSR-LIVE was able to extract a CTA for Mapk1 (Erk2) as well as mTOR, and both were active after 10 min and remained active throughout the experiment (Fig 3A). Activating sites in Mapk1 and mTOR followed a similar temporal response to their CTAs (Fig 3B). In addition, we also identified the CTA of Cdk1, whose substrates decreased in phosphorylation after 45 min, implying deactivation of Cdk1. The authors analysis pointed to mitotic cell cycle as one of the regulated biological processes, which could be controlled by Cdk1 [27]. Although no phosphorylation sites on Cdk1 were present in the NGF Dataset, this could be because these sites were not detected, or Cdk1 activity is inhibited by a mechanism independent of phosphorylation in this context. Nevertheless, both mTOR and Cdk1 have been previously implicated in NGF signaling [34]. These kinases were not revealed in the original analysis, likely because kinase predictions were made based on motifs using NetworKIN [14], which resolves two different kinases only if common consensus motifs are enriched in different clusters of substrates. Our approach overcomes this, by using database knowledge as a starting point and performing clustering considering (the substrates of) each kinase separately. Therefore, the KSR-LIVE analysis tool is a valuable addition to the standard bioinformatics toolkit.

### Novel substrate prediction using KSR-LIVE substrates as training set and ensemble learning

**Fig 4:**
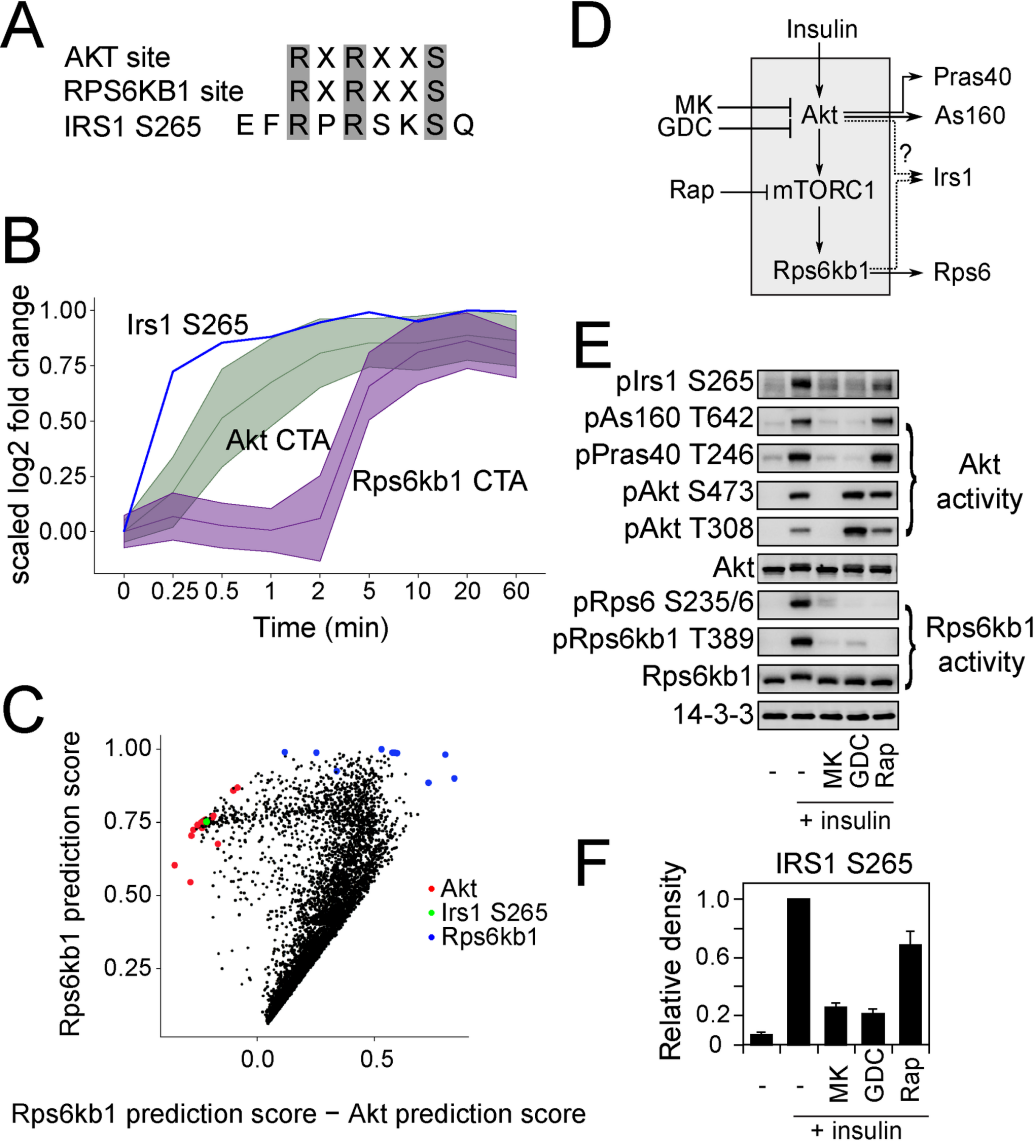
Validation of IRS1 S265 as an AKT substrate. A) Comparison of AKT and RPS6KB1 consensus motif and IRS1 S265 site. B) CTA of AKT (green) and RPS6KB1 (purple) and time profile of IRS1 S265 (blue). (CTA is depicted with mean ± SD) C) Scatter plot of RPS6KB1 prediction scores (y-axis) against RPS6KB1 prediction score—AKT prediction score (x-axis). AKT training substrates are shown in red and RPS6KB1 training substrates are shown in blue. IRS1 S265 is shown in green. D) Insulin signaling via AKT and RPS6KB1. See main text for details. E) 3T3-L1 adipocytes were stimulated with insulin alone or in the presence of inhibitors of AKT (MK, GDC) or mTORC1 (Rapa), after which AKT and RPS6KB1 signaling were assessed by Western blotting. Blots shown are representative of 3 separate experiments. F) Quantification of IRS1 S265 phosphorylation from (E), depicted as mean ± SEM.

In addition to revealing the dynamics of kinase activation, CTAs can also be used to train kinase substrate prediction algorithms, for example the ensemble algorithm described previously [17,35]. To evaluate its utility here we compared the manually curated and automatically generated KSR lists for predicting Akt and mTOR substrates, and found that substrates predicted using either positive training sets was highly similar (correlation coefficient >0.96 for both Akt and mTOR) (S1B Fig). This highlights the utility of KSR-LIVE as a tool for curating a training set for the purpose of predicting novel KSRs using machine-learning methods. We subsequently applied this approach to the kinases Akt and Rps6kb1, because as discussed previously these closely related kinases typically cannot be distinguished by consensus motif based prediction approaches. Firstly, Akt and Rps6kb1 belong to the AGC family and share the same consensus motif (RxRxxS/T [12] Fig 4A). Further, targeted inhibition of Akt also results in attenuated Rps6kb1 activity [13], since this kinase is downstream of Akt (Fig 4D). Despite this, we found that substrates of these kinases can be clearly distinguished by their CTA (Fig 4B), and therefore used KSR-LIVE to provide automatically curated training sets and predict putative novel substrates for Akt and Rps6kb1. We were able to use an ensemble prediction algorithm [17] using the training sets which we were able to acquire for these kinases. Calculating a delta score between the ensemble learning prediction scores for the two kinases (i.e. score difference for Akt and Rps6kb1) readily separated Akt and Rps6kb1 predicted substrates into distinct clusters (Fig 4C). The prediction scores are provided in S2 Table.

We performed leave one out cross validation and achieved a specificity of 0.94+-0.06 for Akt predictions. A candidate substrate of interest was Irs1 S265, which contains an RxRxxS/T motif, and was reported to be an Rps6kb1 substrate [36], but based on its time profile we predicted it to be an Akt substrate (Fig 4B). Thus, we tested our prediction using a panel of inhibitors, consisting of two inhibitors against Akt (MK-2206, GDC-0068) and one against mTORC1 (Rapamycin), which is upstream of Rps6kb1 (Fig 4D). The Akt inhibitors block phosphorylation of both classical Akt substrates (As160, Pras40) and Rps6kb1 substrates (Rps6), whilst Rapamycin completely ablated S6K activity without inhibiting Akt (Fig 4E). The phosphorylation of Irs1 S265 was similarly blocked by both Akt inhibitors, but only modestly reduced by mTORC1 inhibition (Fig 4E and 4F). This implies that Akt plays a predominant role in the phosphorylation of this site after insulin stimulation. In this example, KSR-LIVE was able to automatically dissect Akt and Rps6kb1 activation based on in vivo phosphoproteomics data and together with an ensemble learning algorithm enabled prediction of Irs1 S265 as a biologically-relevant substrate of Akt.

## Discussion

We conclude that using high resolution temporal phosphoproteomics data, KSR-LIVE can dissect phosphorylation signaling within specific biological contexts. KSR-LIVE provides key information on dynamic kinase activation and downstream signaling, and enables the prediction of substrates and biological functions for kinases of interest.

Applying KSR-LIVE to the Insulin Dataset we were able to identify CTAs for 9 kinases. Six of the kinases are activated/inhibited by phosphorylation which follows their CTA with the exception of Mapk1/3. Although the known activating sites on Mapk1/3 are phosphorylated its substrates follow with a time delay of 1 min. This may reflect additional steps in the regulation of this kinase–for instance, phosphorylation of Mapk occurs in the cytoplasm, triggering its nuclear import to target nuclear substrates [37].

Interestingly, for the kinases that are inhibited by phosphorylation, the inverse relationship between kinase and substrate phosphorylation is not maintained for the whole time-course. For Eef2k, S78 phosphorylation decreases with the CTA. Indeed, pS78 coincides with reduced phosphorylation at S392/S396, which is found within the linker site and required for kinases to access S78 [38]. However, S78 phosphorylation inhibits Eef2k activity [31]. Thus, the decrease in the CTA demonstrates that additional regulatory sites, such as S366 (e.g. [32]), maintain the inhibition of Eef2k activity in response to insulin. For GSK3b, the CTA profile increases after 10–20 min whilst kinase phosphorylation is maintained, suggesting factors other than S9 phosphorylation likely affect its activity in the context of insulin action in adipocytes. In summary, the CTA provides insights into the temporal regulation of kinases and the CTA substrates are an effective marker of kinase activity.

We calculated the accuracy of KSR-LIVE by comparing the substrates in the CTA of Akt and mTOR to manually curated gold standard substrates and found that it can achieve an accuracy of over 70% for both kinases offering a significant improvement over the accuracy of the KSR knowledgebase alone. Interestingly, the best accuracy was achieved when the peak of activation was the second time point (30 s for Akt and 5 min for mTOR). Thus, it is crucial to measure phosphorylation at the peak of activation. Studies where the time-course begins after this point will therefore likely miss early activation events, decreasing the accuracy in predicting substrates of early-activated kinases. To achieve highest accuracy it is crucial to study time points that center on the peak of activation of the kinases of interest.

In addition to mapping kinase activity, the extracted kinase CTAs greatly facilitate the prediction of new substrates by providing an automatically generated training set which can be used by learning algorithms, eliminating the resource-intensive and curator-biased process of manually generating a positive training set. Where expert knowledge about substrates is available, manually curated substrates can be added to the automatically generated list, adding more power to the KSR prediction.

The primary advantage of KSR-LIVE over KSR prediction methods using consensus motifs is the consideration of biological context, utilizing biologically relevant KSRs in the training set. This is achieved using time-series data, on the premise that kinase activity can be inferred from the phosphorylation of its substrates. The substrates are not phosphorylated with identical time profiles, due to numerous factors such as copy number, localization and additional regulatory mechanisms, but they may be clustered together to distinguish one kinase CTA from another (Figs 1 and 2). For instance, the Akt CTA rises considerably faster than the Rps6kb1 CTA, enabling us to unambiguously identify the phosphorylation of IRS1 S265 as Akt target even though these kinases share the same motif (Fig 4).

In this context, network inference would be a powerful tool to uncover network topology. However, while the datasets used here possessed many time points and proteome coverage to enable clustering, we could not utilize Bayesian inference because the number of data points was insufficient (data not shown). While other studies have demonstrated this approach is able to elucidate signaling topology, only a small number of phosphorylation sites were considered in their networks [39]–substantially more time points, preferably with several pharmacological or genetic perturbations, would be required to uncover the signaling network on an omic scale.

Generalizing substrate profiles using clustering enables simple and efficient measurement of kinase CTAs (Figs 2 and 3). Once several kinases of interest have been identified, their regulation can be subsequently studied at a mechanistic level. For instance, there are discrepancies between kinase and substrate phosphorylation in the Insulin Dataset; such intricacies may be studied using ordinary differential equation modeling [40,41] with targeted experiments containing focused time-points and inhibitors tailored to the kinases of interest. Thus, KSR-LIVE offers the means to identify patterns in kinase activity that can be subject to further investigation.

The KSR knowledgebase covers a significant portion of the kinome. As it expands, KSR-LIVE will not only be able to assess the activity of additional kinases, but shed more insight into their regulation. For instance, we identified a single cluster for each kinase in the Insulin Dataset, but knowledge of additional KSRs may reveal several substrate clusters for each kinase. Overall, KSR-LIVE has the potential to be included in the standard analysis workflow to study temporal high-throughput signal transduction data. This will further improve our understanding of complex diseases caused by dysregulated signaling, including cancer and type 2 diabetes. KSR-LIVE is publicly available as an R package (**https://cran.r-project.org/package=ksrlive**).

## Materials and Methods

### Integrating databases

The information from four databases were combined into one integrated ‘KSR knowledgebase’. The databases used were: PhosphoSitePlus (retrieved 06/2014), PhosphoELM (release 9.0), PhosphoPOINT (04/2014) and Human Protein Reference Database (release 9). Data from human and mouse was used, and the mouse proteins were mapped to human proteins using the Inparanoid ortholog database (version 8.0). The integrated KSR knowledgebase consisted of 11,666 interactions between 396 kinases and 8,035 phosphosites on 2,431 proteins. Due to redundant UniProt IDs referring to the same protein, Blast (version 2.2.30) was used to map between the integrated database and phosphoproteomics data. The sequences of the proteins were downloaded from UniProt 10/2014.

### Identification of biologically relevant site specific KSRs

Phosphosites with no missing values in two or more replicates were used for clustering. The clustering consisted of two steps, first with only exclusive substrates and in a second step using all available kinase substrates. Both taken together resulted in the final kinase activity marker substrates. {Formatting Citation}

In the first step, substrates exclusive to a kinase were extracted from the integrated KSR knowledgebase and clustered using tight clustering [23]. The user can choose to provide a knowledgebase, see the ksrlive R package for further details. Only exclusive substrates were chosen to assure that the temporal response is the result of one kinase and not multiple ones. The default parameters were used based on the recommendation by Tseng et al. in the tightclust package documentation [42] and the total number of resamplings was set to 100. If a kinase had two or fewer substrates, clustering could not be performed. Background data (points sampled from a uniform distribution in the range of the original data) was added to the exclusive substrates to ensure that if all substrates followed the same time profile they would not be forced apart by the clustering algorithm. The clustered substrates were tested for differential regulation using a 1.5 fold cut off (in two out of three replicates for data from Humphrey et al. and the mean for data from Emdal *et al*.). In the Humphrey et al. data only substrates with multiple replicates were analyzed to assure reliability of the time profiles. The resulting tight clusters formed the core substrates for a kinase. In the second step, clustering was performed using all available substrates for a kinase. All tight clusters containing the original exclusive substrate clusters, were subsequently used to calculate the mean and standard deviation for the characteristic temporal activation profile for the kinase.

### Cell culture

3T3-L1 fibroblasts were maintained in Dulbecco’s modified Eagle’s medium (DMEM) supplemented with 10% (v/v) Foetal Bovine Serum (Life Technologies) and GlutaMAX (Life Technologies) at 37° C with 10% CO_2_. Differentiation was induced at 100% confluence by addition of 250 nM dexamethasone, 350 nM insulin, 0.5 mM 3-isobutyl-1-methylxanthine and 400 nM biotin for 72 h. Cells were then incubated in media containing 350 nM insulin for a further 72 h, and refreshed with naïve media every 2 d after. Adipocytes were used between days 10–12 after initiation of differentiation.

### Assessment of cellular signaling by Western blotting

3T3-L1 adipocytes in12-well plates were washed twice with PBS and incubated with serum-free DMEM containing GlutaMax and 0.2% (w/v) BSA. After 1.5 h, cells were treated for 30 min with 10 μM MK2206 (MK, Selleck Chemicals), 10 μM GDC-0068 (GDC, Selleck Chemicals), 100 nM rapamycin (Rapa, Sigma-Aldrich) or solvent control. Cells were then stimulated where indicated with 100 nM insulin (Sigma-Aldrich) for 20 min before being washed thrice with cold phosphate-buffered saline (PBS) on ice and lysed with 1% (w/v) sodium dodecylsulfate (SDS) in PBS. Lysates were sonicated and subjected to SDS-PAGE and Western blotting as described previously [17]. Antibodies used were against phosphorylated (T308, S473) and total Akt (Cell Signaling Technologies), phosphorylated (T389) and total Rps6kb1 (Cell Signaling Technologies), phosphorylated (T246) PRAS40 (Cell Signaling Technologies), phosphorylated (S235/6) RPS6 (Cell Signaling Technologies), phosphorylated (S265) IRS1 (Santa Cruz), and 14-3-3 (Santa Cruz) as a loading control.

## Supporting Information

S1 Fig Comparison of automatic training set curated by KSR-LIVE and manually curated training set. A) Overlap of Akt (left) and mTOR (right) training sets. B) Scatter plot of prediction scores using the KSR-LIVE training set (y-axis) and the manually curated training set (x-axis). KSR-LIVE training set is shown in blue, the manually curated training set in green and sites that are contained in both are shown in red. Dashed lines represent the top 50 prediction score threshold.
(TIF)

S1 Table Identified kinase substrate relationships. This table lists all substrates that make up the characteristic temporal response of a kinase.
(XLSX)

S2 Table Prediction score for Akt and Rps6kb1. Table of all sites and the kinase prediction score for Akt and Rps6kb1 as well as the sites used as training sets.
(XLSX)

## Abbreviations

KSR: kinase substrate relationship
KSR-LIVE: biologically relevant KSRs within Large-scale In Vivo temporal Experiments
CTA: characteristic temporal activation
