# Correction: Unraveling Kinase Activation Dynamics Using Kinase-Substrate Relationships from Temporal Large-Scale Phosphoproteomics Studies

**DOI:** 10.1371/journal.pone.0185871

**Published:** 2017-09-28

**Authors:** Westa Domanova, James R. Krycer, Rima Chaudhuri, Pengyi Yang, Fatemeh Vafaee, Daniel J. Fazakerley, Sean J. Humphrey, David E. James, Zdenka Kuncic

There are errors in the author byline. The middle initials of the second, sixth, seventh, and eighth authors are omitted. The correct byline is: Westa Domanova, James R. Krycer, Rima Chaudhuri, Pengyi Yang, Fatemeh Vafaee, Daniel J. Fazakerley, Sean J. Humphrey, David E. James, Zdenka Kuncic. The correct citation is: Domanova W, Krycer JR, Chaudhuri R, Yang P, Vafaee F, Fazakerley DJ, et al. (2016) Unraveling Kinase Activation Dynamics Using Kinase-Substrate Relationships from Temporal Large-Scale Phosphoproteomics Studies. PLoS ONE 11(6): e0157763. https://doi.org/10.1371/journal.pone.0157763

Reference 35 is incomplete. The complete reference is: Yang P, Humphrey SJ, James DE, Yang YH. Positive-unlabeled ensemble learning for kinase substrate prediction from dynamic phosphoproteomics data. Bioinformatics. 2016. 32(2):252–9. 10.1093/bioinformatics/btv550.
