# Supplementary figures and images for: Unraveling Kinase Activation Dynamics Using Kinase-Substrate Relationships from Temporal Large-Scale Phosphoproteomics Studies
Source: PLoS One. 2016 Jun 23;11(6):e0157763. doi: 10.1371/journal.pone.0157763 (PMC4918924; doi:10.1371/journal.pone.0157763)

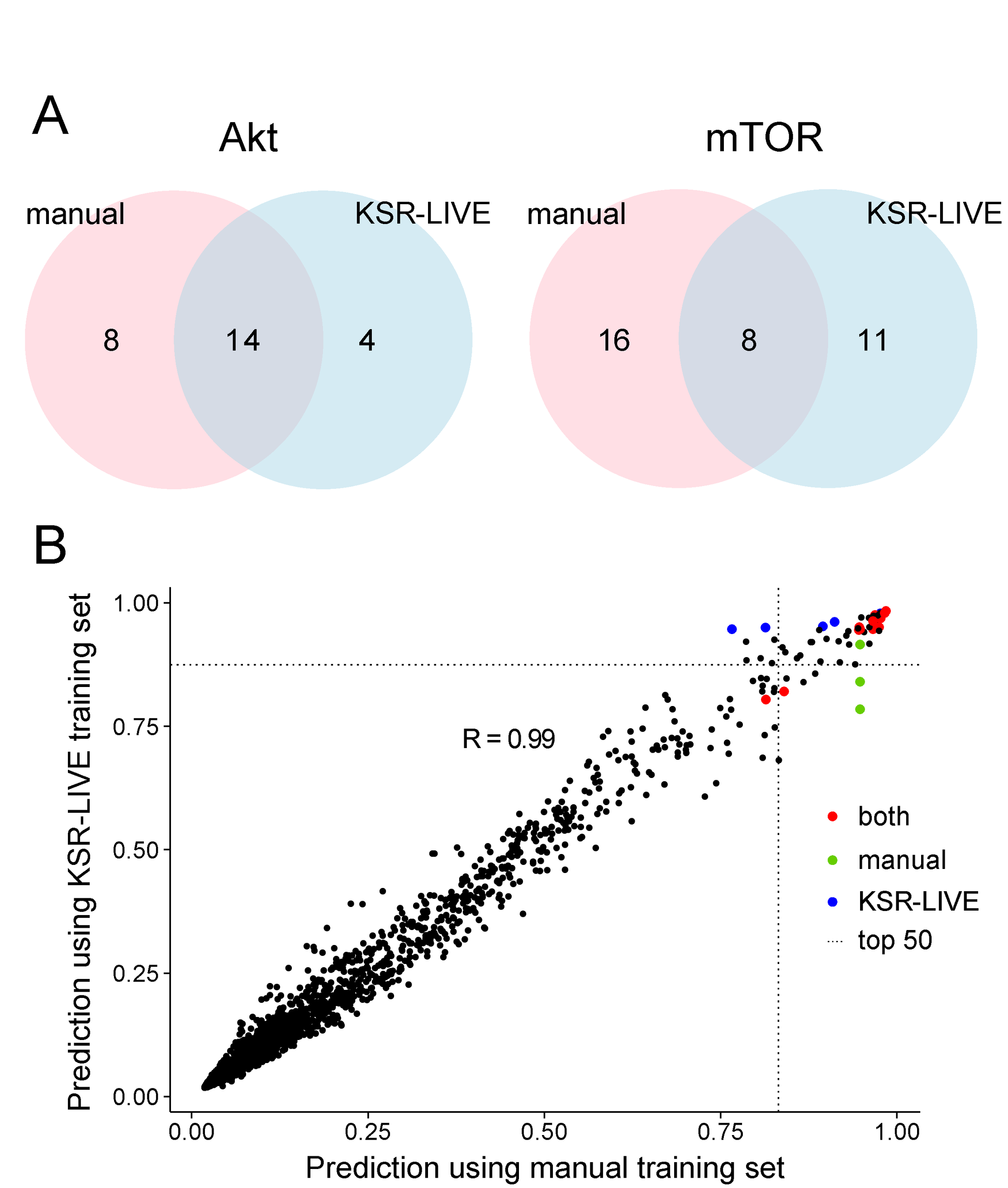

Supplement: S1 Fig — A) Overlap of Akt (left) and mTOR (right) training sets. B) Scatter plot of prediction scores using the KSR-LIVE training set (y-axis) and the manually curated training set (x-axis). KSR-LIVE training set is shown in blue, the manually curated training set in green and sites that are contained in both are shown in red. Dashed lines represent the top 50 prediction score threshold. (TIF) [file pone.0157763.s001.tif]
